# Collaborative research and actions on both sides of the US-Mexico border to counteract type 2 diabetes in people of Mexican origin

**DOI:** 10.1186/s12992-018-0390-5

**Published:** 2018-08-22

**Authors:** Simón Barquera, Dean Schillinger, Carlos A. Aguilar-Salinas, Marc Schenker, Luis A. Rodríguez, Cesar Hernández-Alcaraz, Jaime Sepúlveda-Amor, Mexico-California Diabetes collaborative group, C. A. Aguilar-Salinas, A. Ahued, S. Barquera, S. Bertozzi, A. Bibbins-Domingo, X. Castañeda, J. Cuadros, D. Elias-Lopez, A. Fernández, S. Ponce de Leon, J. Fromow-Guerra, C. Hernández-Alcaraz, M. Hernández, M. Kauffer, D. Kershenobich, L. Muñoz-Hernández, A. Pedroza, J. Rivera, L. A. Rodríguez, J. Salmeron, Laura Schmidt, J. Sepúlveda-Amor, M. Schenker, D. Schillinger

**Affiliations:** 1 Instituto Nacional de Salud Pública, Avenida Universidad 655, Santa María Ahuacatitlán, 62100 Cuernavaca, Mexico; 2 UCSF Division of General Internal Medicine at San Francisco General Hospital, 1545 Divisadero St., First and Second Floors, San Francisco, CA 94115 USA; 3 UCSF Center for Vulnerable Populations, School of Medicine, Department of Medicine, 1001 Potrero Ave, San Francisco, CA 94110 USA; 4 Instituto Nacional de Ciencias Médicas y Nutrición Salvador Zubirán, Avenida Vasco de Quiroga No.15, Colonia Belisario Domínguez Sección XVI, Delegación Tlalpan C.P, 14080 Ciudad de Mexico, Mexico; 5 UC Davis School of Medicine, Department of Public Health Sciences, University of California Davis Medical Sciences 1-C, One Shields Avenue, Davis, CA 95616 USA; 6 UCSF School of Medicine, Department of Epidemiology & Biostatistics, 550 16th St, San Francisco, CA 94158 USA; 7 UCSF Institute for Global Health Sciences, Mission Hall, Box 1224, 550 16th Street, Third Floor, San Francisco, CA 94158 USA

**Keywords:** California, Mexico, Diabetes, Prevention, Global Health, Translational Research, Health Policy

## Abstract

**Background:**

Type 2 Diabetes (T2D) is now a massive epidemic in both California and Mexico, with serious consequences for social and economic well-being. A large proportion of these populations share common ethnic backgrounds. Yet diverse environmental and social conditions across regions create unique opportunities to explore the ways that T2D risk, incidence, management and outcomes manifest.

**Main Text:**

An action-oriented research consortium headed up by the University of California and Universidad Nacional Autónoma de Mexico was constituted to set priorities for bi-national translational research, in an attempt to implement and evaluate clinical, public health and policy actions to decrease the burden of T2D for people of Mexican origin. In this paper, we describe the epidemiology of T2D in Mexico and California, review current efforts to combat the epidemic, highlight gaps in knowledge and identify urgent areas of opportunity for collaboration.

The group has developed a common research agenda and funding has been obtained to evaluate biological samples from the 2016 Mexican Health Survey, collaborate in a telemedicine-based retinopathy project, implement interventions in food banks, promote a communications campaign, and design a large-scale diabetes prevention effectiveness trial.

**Conclusions:**

T2D has caused a state of emergency in Mexico and is a major health problem among Mexican populations on both sides of the border. Understanding the commonalities and differences between California and Mexico for those of Mexican origin with respect to T2D, when combined with a sharing of knowledge and advances, can produce a bi-national translational research agenda to inform relevant policy and practice. Amidst economic and political uncertainty and limited healthcare budgets, this collaboration can contribute to the development of scientific evidence to inform policies and interventions. This may provide a promising collaborative model that could be expanded to other health conditions and regions of the world.

## Background

The American continent is the region of the world with the highest prevalence of obesity and Type 2 Diabetes (T2D), where the patterns of mortality are dominated by non-communicable (e.g. chronic) diseases. A major contributor to the burden of chronic disease in Mexico and the United States of America is T2D [1]. Both countries rank among the top ten countries in the Organization for Economic Cooperation and Development in terms of highest absolute number of cases, and rank first and second, respectively, in terms of population prevalence. The proportion of the population with abnormal glucose tolerance is 52.4% for the US (14.4% T2D, 38% pre-diabetes) [2] and 33.5% for Mexico (14.1% T2D, 19.1% pre-diabetes) [3]. Of note, Mexican-Americans, which constitute the largest ethnic group in the state of California (15 million people, or 38% of the California population) [4] are also the ethnic group with the highest prevalence of T2D in the US (23.8%) [2]. Finally, T2D is a strong risk factor for cardiovascular disease and tuberculosis, doubling and tripling the risk of these comorbid conditions, respectively. As a result, preventing and controlling T2D among people of Mexican origin is one of the many shared and critical interests of Mexico and California, and should be viewed as a bi-national priority.

### Type 2 diabetes in Mexico

While age-standardized death rates per 100,000 inhabitants due to T2D and cardiovascular diseases are similar in the US and Mexico (248.7 vs 199.9 /100,000 inhabitants), T2D mortality is much higher in Mexico (69.2 vs 16.6 /100,000 inhabitants) [5]. Mexico is a nation with one of the highest total mortality attributable to T2D [1, 6]. T2D has been increasing in Mexico since the 1980’s and has become the leading cause of all-age mortality since 2000 [7]. Rapid changes in the food environment, globalization, urbanization and associated lifestyles have been identified as major drivers of this epidemic [8]. Over the past few decades, Mexico has undergone an epidemiologic transition from a country dominated by under-nutrition, infections and high mortality to a country with a somewhat lower overall mortality, but one where diabetes and cardiovascular disease are now the main causes of death [6, 9, 10].

In 2006, only about 6% of people with T2D in Mexico reached a target hemoglobin A1c level of < 7.0% (considered optimal metabolic control) [11]. Six years later, the data from the national survey of health and nutrition show that A1c control has improved to ~ 25% [12]. In 2015, however, the Mexico City Diabetes Representative Survey (MCDRS) found that among the 13.9% of adults with T2D, only 15.8% were under control [13]. Developed countries such as US and Canada have reported glycemic control greater than 50% among their adult population, which although still not ideal, is more than twice the one currently observed in Mexico [14, 15]. Deficiencies of care in Mexico can partly be explained by lack of access to medical services or medication. Approximately 29% of T2D patients are unaware of their diagnosis. The number of medical visits among individuals with diagnosed T2D per year is similar to the international mean, suggesting that patients may be receiving care at an appropriate level of intensity. More than 80% of diagnosed cases are receiving drug therapy.

For several years, efforts have been made to improve outcomes in glycemic control and other endpoints, such as blood pressure, lipid levels, etc. among patients with T2D. However, rates of poor control, complications and mortality remain high. Some chronic complications of T2D (nephropathy, retinopathy and foot problems) are more common in Mexico than in other OCDE countries. In 2013, the Disability-Adjusted Life Years (DALYs) rates due to chronic kidney disease in Mexico increased 136% relative to 1990, with T2D, obesity and hypertension as the main risk factors [6]. Although the incidence of acute diabetes complications has decreased notably, T2D remains as a leading cause of hospitalizations.

Finally, 19% of the Mexican adult population has pre-diabetes, and approximately 40% has metabolic syndrome (a strong risk factor for T2D and cardiovascular disease), defined as a conglomerate of risk factors including central obesity, elevated blood glucose, triglycerides and blood pressure, and/or reduced HDL cholesterol [16, 17]. Because T2D progresses from the overlapping conditions of metabolic syndrome and pre-diabetes, efforts to prevent T2D invariably will need to focus on the large subset of the population (~ 40% of adults) with these conditions.

### Diabetes in Mexican-Americans

As described above, adults of Mexican origin living in the US, when compared to other racial/ethnic groups in the US, have one of the highest prevalence of diabetes (23.8% compared to 11.3% in non-hispanic white), and have rates much higher than Mexicans residing in Mexico [2]. In California, an additional 44% of Latinos have pre-diabetes [18]. Many US studies report an increased risk among Latinos in general, and Mexican-Americans in specific, both with respect to developing T2D and disproportionately suffering some of its complications. There appears to be a particular vulnerability to micro-vascular complications, such as retinopathy and blindness; nephropathy and end-stage kidney failure; and neuropathy and associated amputations. Diverse factors have been hypothesized to underlie these vulnerabilities, including genetic susceptibility, perinatal conditions including malnutrition and breast-feeding practices, adverse dietary and lifestyle patterns related to acculturation (high consumption of sugar-sweetened beverages, processed foods and low physical activity), food insecurity and lower socioeconomic status, poor access to health services, receipt of poor quality healthcare services, communication barriers (such as limited literacy and limited English proficiency) [19, 20] and sub-optimal adherence to treatment recommendations.

One of the most serious concerns now facing the Mexican-American community in California is the “greening” of the T2D epidemic; once considered a disease of the old, T2D is now affecting youth and young adults. The mean age at time of T2D diagnosis among Hispanic Americans in the US is now less than age 50 [21]. Whereas only 15 years ago fewer than 1 in 10 US teens had pre-diabetes, now approximately 1 in 5 do [22]. Rates are even higher for Mexican-Americans; in fact, nearly 1 out of 100 Mexican-American teens (0.9%) now has diabetes. The Centers of Disease Prevention and Control has projected that a Mexican-American child born in 2000 has a greater than 50% chance of developing T2D in their lifetime [23].

### Current national efforts against T2D in Mexico

Mexico and the US have begun to identify T2D as major threats to economic and social well-being. Recently the Mexican government declared a “state of emergency” due to the high incidence, poor prognosis and rise in diabetes mortality [24]. Multifaceted strategies have begun to be implemented in both countries, but remarkable differences exist in their approaches for the prevention and control of T2D.

A number of national prevention policies have been implemented to curb the T2D epidemic in Mexico. Some of the more notable ones are 1) the “5 Pasos” (Five Steps) national program in the 2006–2012 administration, which promotes early detection, increase in physical activity, potable water for hydration, reduction in caloric consumption and supporting community and family engagement, 2) the national healthy hydration recommendations, 3) healthy nutrition guidelines for the school environment, 4) a national agreement for healthy nutrition (2010), which subsequently became a national policy (2013), 5) the “Chécate, mídete, muévete” (Check yourself, test yourself, and move) national program in the current administration, which promotes healthy lifestyle behaviors and early detection of T2D and 6) a national 10% excise tax on soda and sugar-sweetened beverages, which has been demonstrated to yield a substantial reduction in consumption and potential health benefits [8, 25–28]. Other areas, such as implementing a nutritional front-of-pack labeling system and marketing regulations are priorities which are yet to be undertaken, and will require support from the Ministry of Health and academic groups free of conflicts of interest with the food industry [29, 30]. Recently, the Mexican National Academy of Medicine has produced two influential position papers on the topics of obesity and diabetes with recommendations to curb the current T2D epidemic [31, 32].

In addition, innovative models of healthcare for T2D patients have emerged; several Mexican health systems have implemented prevention and treatment strategies to increase effectiveness while lowering costs. Examples are the DiabetIMSS program (by the Instituto Mexicano del Seguro Social), the UNEMES Crónicas (by the Health Ministry) [33], MIDE (by the Instituto de Seguridad Social y Servicios para los Trabajadores del Estado) [34] and CAIPADi (by the Instituto Nacional de Ciencias Médicas y Nutrición) [35]. While some of these clinical programs focus on empowerment of patients and their relatives, others provide easy access to diabetes educators and multidisciplinary teams. Despite some achievements in the aforementioned programs, major efforts are still needed to improve quality of care in primary care units nationwide. Private partners have also become involved. For example, the “*Casalud*” program, financed by the Carlos Slim Health Institute/Foundation, offers a model that uses technology (mobile apps and SMS messages) to deliver T2D prevention and care. *Casalud* has established partnerships with several Mexican state governments to deploy the model nationwide. Preliminary efficacy evaluations of these clinical interventions are promising and have been reported (http://oment.uanl.mx). In addition, the Mexican Academy of Medicine presented a set of recommendations to impact several processes involved in the prevention and care of T2D [32].

Efforts to develop hard-hitting social marketing campaigns to prevent T2D are at an early stage, but appear promising. The initiative *Destapa La Verdad*, coordinated by El Poder del Consumidor (a consumer rights advocacy organization) and a number of public health and nutrition partners (http://destapalaverdad.mx), which has targeted the reduction in consumption of sugar-sweetened beverages, recently partnered with youth and young adult musician-activists to create the CD, *Dulce Veneno*. Modeled off of a successful California-based campaign (see below), this effort attempts to [1] engage young people as authors of social transformation and [2] promote changes in social norms to prevent T2D in young people.

### Major challenges, however, remain unaddressed

Multi-sector coordination of initiatives is urgently needed. Urban planning, education, healthy food availability, access to clean water and physical activity spaces require coordinated efforts to tackle this health challenge effectively. In addition, national registries and translational research are currently at an early stage. Four major challenges need to be addressed: first, reduce exposure to the factors that increase risk for T2D at the population level; second, increase identification and diagnosis of patients with pre-diabetes and diabetes; third, increase effectiveness of therapeutic interventions to meet or exceed international standards of control; and fourth, increase early identification and control of complications to improve the quality of life of population with diabetes [32]. Despite the remarkable social and economic cost of T2D chronic complications (especially diabetic retinopathy and end-stage kidney failure), there is little information at the level of the Mexican healthcare system regarding the quality and processes of care in both the prevention of quality of care for retinopathy and kidney failure.

### Interventions targeted to populations of Mexican origin in the US

Federal, state, and local immigration policies, as well as the political climate around immigration, are important determinants of health among the Mexican-origin community in the US. While recent studies have found that undocumented immigration does not increase violent or non-violent crimes, the Trump administration’s rhetoric towards Latin-American and non-European immigrants, including labeling them criminals and predators, has created a climate of fear among immigrants, is accelerating the deportation of undocumented immigrants, and contributes to structural racism. A recent review of federal- and state-level immigration policies identified the following four mechanisms for how immigration policies impact Latino health, including the health of those of Mexican origin: 1) stress related to structural racism and discrimination; 2) reduced access to beneficial social institutions and the social safety net, especially education; 3) worse access to healthcare and related services; and 4) constrained access to material conditions such as food, wages, working conditions, and housing [36]. For instance, compared to non-Hispanic whites, there is a lower SNAP participation rate (previously known as “food stamps”, a governmental program to mitigate food insecurity) among eligible immigrant minorities, in part due to fear of deportation or interference with their efforts to acquire citizenship [37–39]. Food insecurity is a strong risk factor for incident T2D [40] and its complications. . In addition, problems accessing care affect the entire T2D spectrum, from prevention to control to the management of complications.

While not solely intended as a policy-level intervention to reduce the burden of T2D among Mexican-Americans, the Affordable Care Act (ACA) of 2010 (“Obamacare”) has made early diagnosis and management of T2D more accessible for Mexican-Americans in California. The first five years of the program yielded a reduction in the uninsured Hispanic-Americans from 26 to 16%. By comparison, among non-Hispanic whites, rates fell from 14 to 10% over the same time period. Extensive efforts have been expended in California to successfully enroll Mexican-Americans in health insurance under the ACA [41]. To date, we are not aware of evaluations of the ACA on T2D-related outcomes among Mexican-American populations.

Since 1980, the US has implemented the National Diabetes Surveillance System, which has been documenting the public health burden of T2D and its complications. Starting in 1997, the CDC began to report data on US Hispanic populations, including Mexican-Americans [42]. More recently, in 2010 the US Congress authorized the CDC to establish the National Diabetes Prevention Program. This program, which is an individual-level program (in contrast to Mexico’s initiatives that focus on structural interventions), revolves around implementing research findings of the National Institutes of Health-funded Diabetes Prevention Program (DPP) in communities across the US [43]. The DPP, published in 2002, found that engagement with a rigorous program to promote physical activity and improve dietary intake yielded a 58% reduction in incident diabetes, among diverse individuals with pre-diabetes. Of note, DPP also found that twice-daily Metformin reduced incident T2D by 31% relative to control. The National DPP, in an attempt to translate the efficacy study and promote uptake, created a certification process for community-based and on-line programs to deliver a CDC-recognized lifestyle change program to prevent or delay T2D. National DPP is a partnership of public and private organizations working to reduce the growing problem of pre-diabetes and T2D. All states now have such certified programs, although reimbursement for patients is highly variable, and few programs have specifically targeted Mexican-Americans. However, the CDC hosts an online repository and registry of certified programs at the local level, although language access information is lacking [44].

In California, there has been a slightly greater emphasis on structural interventions compared to the rest of the US. An influential report from the California Center for Public Health Advocacy advocated for community and workplace environments that support healthy eating, and promoting built environments that encourage physical activity [18]. In addition, California has been home to a number of statewide programs. In 2013, a *Prevention First* Initiative was awarded funding from the CDC to prevent and control diabetes, heart disease, obesity and associated risk factors and promote school health. Objectives include increasing availability of free drinking water at schools, increasing the percentage of schools that prohibit non-essential food advertising, augmenting physical activity programs at child care facilities and schools, and increasing the number of Baby-Friendly labor and delivery facilities that promote breast-feeding [45]. However, we are not aware of any statewide program that specifically targets Mexican-Americans or Hispanic Americans.

In 2010, the US Centers for Disease Control and Prevention (CDC) created the National Program to Eliminate Diabetes-Related Disparities in Vulnerable Populations, a five-year cooperative agreement (2010–2015). The main goal of the agreement was to identify and carry out public health activities specific to the culture of selected communities to address differences in health outcomes associated with diabetes. These activities aimed to prevent and control factors that increase the risk for developing T2D and its complications, such as poor nutrition and physical inactivity; raise T2D awareness; promote diabetes self-management; and improve outcomes and quality of life for those living with T2D in high-risk communities and regions across the country. The National Alliance for Hispanic Health was a recipient, and a demonstration project was recently completed in Watsonville, CA, an agricultural region. Results are forthcoming.

The DPP focused on weight loss interventions based on reducing energy intake and dietary fat and increasing physical activity. However, complementary interventions to decrease added sugar intake, particularly from sugar-sweetened beverages are largely lacking. This is a particularly important area of opportunity among high-risk Mexican-American populations due to their elevated consumption rates of added sugars and sugar-sweetened beverages (SSBs), and their susceptibility to developing T2D and its microvascular complications (kidney failure, blindness, neuropathy and amputations). Between 2005 and 2010, US adults consumed approximately 13% of total energy from added sugars, with similar intakes between non-Hispanic whites and Mexican-Americans [46]. However, among youth in California, compared to whites, Latino children consume greater amounts of juice, and Latino adolescents consume more soda [47, 48].

In general, in the US, about 33% of added sugar intake came from SSB’s. However, in contrast to the US, the majority (~ 69%) of added sugars consumed by Mexicans in Mexico are in the form of SSBs [49]. Globally, Mexico ranks highest in absolute and proportional deaths attributable to SSBs consumption [50]. Modeling studies have found reductions in SSB consumptions to produce important reduction in obesity, T2D and coronary heart disease among Mexicans. Similarly, in the US, reductions in SSB consumption among Mexican-Americans would generate disproportionately greater benefits compared to other US racial/ethnic groups [51]. The root causes of the disproportionate impact that SSBs play on the health of Mexicans in the US and Mexico is likely multifactorial, and includes (a) limited health literacy/lack of awareness of untoward health effects, (b) targeting and unfettered marketing of SSBs to these communities, (c) the addictive and properties of liquid sugar, particularly in the face of poverty and stress, (d) the integration of SSBs into individual, family and communal social norms, and (e) concerns about water safety on both sides of the border, particularly in rural areas and lower-resourced urban and suburban settings, and the high relative cost of bottled water vs. SSBs [51].

However, there have been regional and municipal health promotion campaigns and regulatory efforts to reduce population exposure to SSBs. Many county health departments host media campaigns, such as the Open Truth Campaign, to promote water and reduce SSB consumption. In 2015, the City of Berkeley, California was the first governmental entity in the US to approve a tax on SSBs, and the one-year evaluation demonstrated dramatic reductions in SSB consumption, as well as concomitant increases in water consumption [52]. Three other municipalities in the San Francisco Bay Area passed similar measures in the fall 2016. Relatedly, in 2015, the City and County of San Francisco was the first in the world to pass an ordinance requiring that outdoor advertising of sugar drinks contain a warning notice related to the risks of obesity, diabetes and tooth decay. The American Beverage Association sued the City in a federal constitutional court case related to freedom of speech; the City won that case, but implementation has been delayed until the outcome of a judicial appeal [19].

As with Mexico, California is home to an innovative social marketing campaign that brings together the Arts with Public Health to engage at-risk youth in changing the social, environmental and structural conditions that are the root causes for the T2D epidemics in their communities. *The Bigger Picture Campaign* (www.thebiggerpicture.org) harnesses spoken word poetry to tap into two of the most important values of adolescents – defiance and social justice – to motivate change [53]. This campaign, which has created 24 powerful on-line videos, has received numerous public health and film awards and has reached over 1.5 million people. Evaluations of the Bigger Picture have been very promising [54]. Finally, there now exists a small body of US research regarding successful clinical and community programs that have been developed de novo or culturally adapted to meet needs of the Mexican-American population with T2D, including addressing barriers related to language, culture, food insecurity, heath literacy and access to T2D specialty care [55–61]. However, there has been little effort to scale up such effective programs, a relative paucity of research on prevention, and relatively little to no attention on policy-level intervention research specific to this high-risk group. Consequently, experiences from both sides of the border may be helpful to informing and implementing primary and secondary preventive programs related to T2D for populations of Mexican origin.

### Leveraging binational efforts to study T2D prevention and control

A greater commitment to translational research is needed to help authorities and stakeholders develop plans for future action. Research is critical to plan and implement policies to combat T2D. However, since the US-Mexico Border Project ended, there has been a lack of coordinated bi-national efforts to improve our understanding of the epidemic in this population.

There are a number important research opportunities for the study of prevention and control of T2D in Mexicans living in Mexico and California. The uniqueness and diversity of the populations and their respective environments can be harnessed to generate new local and global knowledge. For example, Mexicans have a higher proportion of Amerindian heritage (30–50%), a group that is inadequately represented in the genetic consortia [62]. This ethnic group develops T2D at a younger age and with a lower BMI than that found in individuals of European descent. In addition, studying their increased susceptibility to microvascular complications (especially diabetic nephropathy and retinopathy) in samples of individuals with the similar genetic backgrounds who are living in different settings, can offer insights into the pathogenesis of T2D-related complications. Environmental risk factors related to T2D (e.g, migration, exposure to pollutants or calorie-dense foods and beverages) have evolved rapidly in the region. [63]. Public policies against T2D could be tested in a shorter period of time than in other areas because of the large number of outcomes related to T2D. In addition, low-threshold interventions could be implemented in populations of the same ethnic origin but living in different socioeconomic contexts. Additional research and development of innovative health communication interventions, such as *The Bigger Picture* and *Dulce Veneno*, is a pubic health priority, and collaborative exchange between such campaigns could yield new insights and generate additional resources to scale these programs. Little is known about risks for special sub-groups (e.g. gestational diabetes, pre-diabetes, monogenic forms of diabetes and for most chronic complications), including insufficient data for many of the outcomes related to T2D (including DALYS and quality adjusted life years (QALYs). The use of low-threshold interventions to prevent T2D and its complications is at an early stage. Interventions for preventing T2D that target patients with certain characteristics have been developed, but their use has been limited to efficacy studies.

Two of the National Institutes of Health of Mexico, the National Institute of Medical Sciences and Nutrition (INNSZ) and the National Institute of Public Health (INSP), have collaborated for several years to describe the epidemiology, genetics and the use of health services of patients with T2D. Moreover, several campuses of the University of California are leaders on various issues related to T2D (e.g., translational research, genomics, gestational diabetes, prevention programs). A platform for collaborative research between Mexico and the University of California can enhance ongoing institutional and governmental efforts and create lead to discoveries related to T2D in populations of Mexican origin. In November 2015, a group of researchers from the University of California, the Mexican National Institutes of Health (represented by the INNSZ and INSP), the Mexican National Autonomous University (UNAM) and the Mexican National Institute of Social Security held a joint meeting at the UC Institute of Global Health to discuss possible collaborations to respond major common health problems. T2D was selected as one of 4 focus areas of this UC Mexico Health Initiative. In April of 2016, this T2D group had a first meeting in Mexico City to develop an agenda of collaboration and identify and design a multi-stage multidisciplinary research program on T2D. This program is focused on both understanding the complex origins of the T2D epidemic among people of Mexican descent and identifying practical and effective solutions to improve prevention and control of the epidemic. Creating a common, coordinated agenda is valuable for many reasons. First, economies of scale may be created by the synergies between the translational research groups. Second, researchers will benefit from multidisciplinary international exchange. Third, having the largest public universities in both countries (UC and UNAM) supporting this initiative can also generate political support, educational opportunities, financial resources ad other forms of capacity that might not be possible otherwise, or could take a much longer time to develop.

### A proposed research agenda for T2D prevention and control in Mexicans and Mexican-Americans

The consensus objective of the bi-national T2D group is to reduce the health burden of T2D on society in Mexico and California by focusing on the study of prevention and control strategies for populations of Mexican origin. Examples of such collaborative work include successful cross-national work on tuberculosis control, tobacco control and HIV control/AIDS prevention [64]. Our underlying principles include collaborative team-work, promoting public health literacy, maintaining reliable and ongoing sources of data for surveillance and evaluation, prioritizing and evaluating clinical and cost-effectiveness of interventions, and ensuring that interventions are scalable, can have a strong population-level impact, and are independent from (diabetes- and nutrition-related) industry interests. Finally, we focus our efforts on vulnerable populations, defined as sub-groups of the larger population that carry a *greater risk of risks* due to social, economic, environmental, historical or political forces.

The Diabetes subgroup of the UC Mexico Health Initiative is taking a sequential approach to address the T2D epidemic among the Mexican-origin populations in Mexico and California. Three sequential yet overlapping phases are being developed. The first phase started in late 2015 with the creation of the subgroup, identification of its co-chairs and teams, development of its mission, objectives and goals and creation of essential descriptive studies. Phase II began in mid-2016 and is primarily focusing on the discovery of new knowledge by carrying out population-based surveys (ENSANUT 2016), collection of biometric data, developing modeling studies of potential benefits of interventions based on the Mexican and American CVD Policy model, synthesizing the promise of current and future T2D prevention and control policy efforts, and influencing the public discourse related to food and beverage policy. In addition, a matrix of Mexican and Californian researchers was built to enhance the interactions between participating institutions. Phase III will begin in 2017 and will attempt to develop medium- to large-scale interventions with potential to make a significant impact on the T2D rates and improve the health of patients suffering from T2D. These include designing an intervention to harness the available “Social Safety Net” to prevent T2D (e.g. food banks in US and social programs such as *Oportunidades* in Mexico), implementing a quasi-experimental trial using Metformin for T2D prevention, and developing an initiative to detect and reduce eye and kidney disease. These interventions will run in parallel with the implementation of communication campaigns to enhance awareness, disseminate results of programs and gain broader public attention and support.

As of December of 2016, the group has made progress on the short/intermediate objectives, as well as initiating some of the long-term objectives. The group has created a matrix of translational researchers at UC and Mexico. A modeling study exploring the impact of the Mexican soda tax on diabetes and cardiovascular health and health expenditure has been recently published [28] and a cost-effectiveness analysis of a modeled intervention of metformin vs. lifestyle for the prevention/delay of onset of T2D among Mexican adults in Mexico with pre-diabetes is in progress. It has also authored influential papers demonstrating (a) the extent of bias in experimental etiologic research related to diabetes pathogenesis resulting from the financing of science by the sugary beverage industry [65] and (b) a critique of the food and beverage industry’s claims that WHO’s added sugar guidelines are not evidence-based [66]. The group also obtained a grant to evaluate biological samples from the Mexican National Health and Nutrition Survey (ENSANUT 2016), blood sugar, lipids and for the first time indicators of kidney disease. It has also obtained funding to continue the development of an early detection system for retinopathy in Mexico, taking advantage of the telemedicine experience developed in California [55], and is carrying out a funded diabetes prevention and control intervention in food banks in California. Finally, both UC and Mexico teams have met with the Secretary of Health of Mexico City, and are in the planning phases of a large primary care-based effectiveness trial of Metformin plus lifestyle for T2D prevention in Mexico City’s public healthcare system.

## Conclusions

T2D is the major health problem in Mexican population on both sides of the border. The prevention and control of T2D among people of Mexican origin requires extensive knowledge of the multidimensional causes of the disease, and development of evidence-based investments in multi-sectorial, multi-level efforts involving feasible and cost-effective interventions. In the context of geographic proximity and common ancestry, the existence of a large population of Mexican origin with differential exposures to unhealthy environments, economic stressors, and limited public health budgets, combined with the urgent need to implement policies and actions, a joint bi-national collaboration between California and Mexican health researchers might be one essential component to successfully tackling the T2D epidemic by improving our understanding of feasible and effective interventions.

There is an important gap between the quality and quantity of information generated by those in academia and the implementation of effective interventions into practice in real life settings, particularly among vulnerable populations. The combined resources in low to middle income countries and higher income countries should be leveraged to reduce this gap. The development and nurturing of collaborative groups between countries – collaborations that involve community- and policy-engaged researchers - is one option to confront this challenge. The need to understand susceptibility to disease in populations with common ancestry is also easier to approach in a context of collaboration, where exposure to different environments can shed light on vulnerability and resilience, as well as highlight avenues for intervention.

Unlike other health problems, the prevention and control of non-communicable chronic diseases such as T2D will not only require the development of high-tech/low cost healthcare interventions but also on policy-level actions. For example, national policies such as the front-of-pack warning labels for unhealthy food in Chile and the soda tax in Mexico are being imported by developed countries attempting to prevent and control chronic diseases [67, 8]. Such examples suggest that mutual benefits are likely in these types of collaborations.

The context of globalized economies, diverse migration patterns, evolving anti-immigrant policies and their associated health challenges, together has created an increasing complex environment requiring multi-national cooperation to improve the health of populations. Although the example of the T2D epidemic in people of Mexican origin in the US and Mexico in some ways is unique, the processes and products developed by this binational collaboration might prove to be a successful model and case study for countries sharing similar burdens of disease in an increasingly globalized world.
